# Editorial: Experimental models of asthma

**DOI:** 10.1186/1745-6673-3-S1-S1

**Published:** 2008-02-27

**Authors:** Armin Braun, Thomas Tschernig, David A Groneberg

**Affiliations:** 1 Fraunhofer Institute of Toxicology and Experimental Medicine, D-30625 Hannover, Germany; 2 Department of Anatomy, Hannover Medical School, D-30625 Hannover, Germany; 3 Institute of Occupational Medicine, Charité – Universitätsmedizin Berlin, Free University and Humboldt-University, D-13353 Berlin, Germany

## Abstract

Since 2002, a workshop entitled “Asthma in animal models” has been held once a year in Hannover, Germany. It is organized by the Fraunhofer Institute of Toxicology and Experimental Medicine in collaboration with the collaborative research centre “Sonderforschungsbereich” 587, “Immune reactions of the lung in infection and allergy” (Hannover Medical School). The aim of these meetings is an intense scientific exchange between researchers and clinicians coming from academic or industrial background. Over the years the topics within the extensive field of asthma and COPD have ranged from methodological aspects to the influence of infections and environmental factors up to perspectives in the development of new therapeutic strategies.

## Editorial

In January 2007, the 6th workshop took place with the main topics “Interaction of resident lung cells with inflammation in asthma and COPD”, “Infection and Asthma” and “New therapeutic Targets against Asthma”. The workshop was organized by the Fraunhofer Institute of Toxicology and Experimental Medicine in collaboration with the collaborative research centre “Sonderforschungsbereich” 587, “Immune reactions of the lung in infection and allergy” (Hannover Medical School). Some particularly interesting contributions to the workshop were selected to be published in this supplement to *Journal of Occupational Medicine and Toxicology* since inflammatory airway diseases are of major interest for the research areas covered by the journal [1-9].

Bronchial asthma is a respiratory disease characterized by chronic inflammation and episodes or attacks of airway narrowing. The burden from asthma in western societies has increased over the past decades, but the precise reasons for this increase remain enigmatic so far. In addition, the exact physiological mechanisms of the development of asthma, in particular the complex interaction of chronic inflammation with resident lung cells such as smooth muscle cells, fibroblasts or neurons are still unknown.

To understand the disease and subsequently develop new and effective drugs, the use of predictive animal models is essential. Since several newly developed highly specific anti-inflammatory drugs were very effective in murine asthma models but failed to be effective in patients, the discussion about the adequate animal models emerged.

Bronchial asthma is still a major socioeconomic burden, that is, 17 million US Americans, with approximately one third of those being children, were affected by bronchial asthma in 1996. Also, the economic burden of the disease is increasing in parallel, from US$6.2 billion in 1990 to an estimated US$12.7 billion in 1998 [10]. Similar trends are reported from the Asian region: based on prevalence rates at present and projected increases, it can be estimated that the total population of Chinese asthmatic patients will be around 150 million individuals by the year 2013. This dramatic numbers includes 38 million children [11]. With regard to the spiraling treatment costs, it is important to debate that there should be a substantial realignment of drug development policy in the pharmaceutical industry and a parallel shift in the licensing policy by authorities to encourage the development of novel compounds and substance classes that are effective in halting the progression from acute to chronic forms, when the disease first manifests in early childhood [12]. These new agents include both substances that represent improvements of existing drug forms, such as inhaled steroids or inhaled beta 2 receptor agonists and completely new classes of drugs. These new classes include agonists and antagonists of specific extracellular and intracellular mediators such as neuromediators, cytokines or chemokines as well as modulators of immune responses like toll like receptor agonists.

It is also important to mention that the pharmacologic treatment options are not curative. Current and future approaches should lead to a better long-term control of chronic airway obstruction. In this respect, it should also be a major aim to develop novel agents that are as effective existing drugs but have less side effects and have a better route of administration [13].

This supplement to *JOMT* aims to provide a series of reviews that focus on current knowledge in pathogenesis and evolving therapeutic options in allergic asthma.

The series starts with an article about the “Role of mast cells in asthma” [14]. Mast cells are known for long time to play an important role in the pathophysiology of allergic diseases. Therefore, blocking of mast cell mediators like histamine is a very commonly used treatment option in e.g. allergic rhinitis. However, in asthma the role of mast cells is less well defined and antihistaminic drugs are less effective. First experiments in mouse models lacking mast cells could not prove an significant role of mast cells in the development of allergic asthma [15]. Reuter and Taube describe in their review that this is due to the unphysiological sensitization of the animals using adjuvant. In contrast, in protocols using less potent sensitization procedures, the role of mast cells for induction of allergic airway disease can be proofed. The review gives detailed insights in the mechanisms of mast cell contribution by describing the function of the released mediators like LTB4 and TNF-alpha.

The series continues with an article describing the interaction of allergic airway disease with infections [16]. On the one hand side lung infections are known to be classical trigger factors of asthma exacerbations, on the other hand, exposure to microbial products is known to be protective against allergic asthma. In addition, Beiswenger and Bals describe that allergic airway inflammation also inhibits antimicrobial host defense and renders animals more susceptible to bacterial infection. The paper elucidates the complex interaction between allergic inflammation and microbial infection.

A further focus addresses strategies for the identification of new therapeutic targets in a chronic asthma model [17]. This review describes the development of improved models of allergic asthma using repeated allergen aerosol challenges over several weeks resulting in profound and sustained allergic airway inflammation, airway hyperresponsiveness and airway remodelling. Wegmann describes here how he has used this model to test new compounds like a low molecular weight antagonist of CCR-3. Special focus is given on the role of T-cells in allergic inflammation and the Th-2 promoting transcription factor GATA-3 is introduced as an interesting target for further intervention.

The contribution by Fuchs deals with the function of TLR-2 agonists in allergic lung inflammation [18]. In this paper, the biology of TLR-2 is described and the function of this receptor in innate and adaptive immune response of the lung is highlighted. TLR-2 is of particular interest since genetic variation in TLR2 is a major determinant of the susceptibility to asthma and allergies in children [19]. In animal models the immune modulatory capacity of TLR-2 agonists like MALP-2 is demonstrated. However, for effective treatment of experimental asthma the addition of IFN-gamma is augmenting treatment success.

The last two contributions of the supplement provide an analysis of novel mechanisms of inflammation [20] and scientiometric data [21].

Taken together, this supplement gives insights in the actual discussion regarding animal models of asthma and shows some examples of how they are used to elucidate the pathophysiology of the disease and develop new therapeutics against asthma.
